# Index admission cholecystectomy for acute cholecystitis reduces 30-day readmission rates in pediatric patients

**DOI:** 10.1007/s00464-023-10632-7

**Published:** 2023-12-19

**Authors:** Sagar J. Pathak, Hyun Ji, Amar Nijagal, Patrick Avila, Sun-Chuan Dai, Mustafa A. Arain, Abdul Kouanda

**Affiliations:** 1 Division of Pediatric Gastroenterology, Department of Pediatrics, University of California San Francisco, San Francisco, CA USA; 2 Division of Gastroenterology, Department of Medicine, University of California, San Francisco, San Francisco, CA USA; 3 Division of Gastroenterology, Department of Medicine, Center for Interventional Endoscopy, AdventHealth, Orlando, FL USA; 4 Department of Surgery, University of California, San Francisco, CA USA; 5 Liver Center, University of California, San Francisco, CA USA; 6 The Pediatric Liver Center, UCSF Benioff Children’s Hospital, San Francisco, CA USA; 7 Eli and Edythe Broad Center of Regeneration Medicine, University of California, San Francisco, CA USA

**Keywords:** Pediatric, Hepatobiliary, Surgery, Outcomes

## Abstract

**Background:**

Adult patients with cholecystitis who do not undergo cholecystectomy on index admission have worse outcomes, however, there is a paucity of data of the role of cholecystectomy during index hospitalization in the pediatric population. Our aim was to determine outcomes and readmission rates among pediatric patients with cholecystitis who underwent index cholecystectomy versus those who did not.

**Methods:**

We performed a retrospective study of pediatric (< 18 years old) admitted with acute cholecystitis (AC) requiring hospitalization using the 2018 National Readmission Database (NRD). Exclusion criteria included age ≥ 18 years and death on index admission. Multivariable logistic regression was performed to identify factors associated with 30-day readmissions.

**Results:**

We identified 550 unique index acute cholecystitis admissions. Mean age was 14.6 ± 3.0 years. Majority of patients were female (*n* = 372, 67.6%). Index cholecystectomy was performed in (*n* = 435, 79.1%) of cases. Thirty-day readmission rate was 2.8% in patients who underwent index cholecystectomy and 22.6% in those who did not (*p* < 0.001). On multivariable analysis, patients who did not undergo index cholecystectomy had higher odds of 30-day readmission than those who did not (OR 10.66, 95% CI 5.06–22.45, *p* < 0.001). Female patients also had higher odds of 30-day readmission compared to males (OR 3.37, 95% CI 1.31–8.69).

**Conclusions:**

Patients who did not undergo index cholecystectomy had over tenfold increase in odds of 30-day readmission. Further research is required to understand the barriers to index cholecystectomy despite society recommendations and clear clinical benefit.

**Supplementary Information:**

The online version contains supplementary material available at 10.1007/s00464-023-10632-7.

The incidence of acute cholecystitis has been steadily increasing in children over the last several decades emphasizing the need for further study and consequent development of evidence based-guidelines [1–3]. Children most at risk for acute cholecystitis are those with comorbid conditions that would increase their propensity to form gallstones such as hemolytic disorders, obesity, congenital malformations, parenteral nutrition, cystic fibrosis, and short bowel syndrome [1, 4]. Early cholecystectomy in adult patients with acute cholecystitis has been widely studied and shown to be safe with less morbidity, shorter hospital stays, and lower readmission rates than those who underwent delayed cholecystectomy [5–9]. In contrast, ideal surgical timing and management of the pediatric population has been less studied, which has led to heterogenous management practices [10].

A retrospective study by Diez et al. evaluated the management of pediatric versus adult cholelithiasis within their institution [10]. This study noted a preferred conservative approach in the pediatric population compared to the adults with noted increased median time of symptoms (4 vs 1 months, *p* = 0.075) and time from diagnosis to surgery (15 vs 4 days, *p* = 0.128). During an online questionnaire, the group found that 22% of participants reported conducting a cholecystectomy within 24 hours of symptoms, 4% reported conducting one within 48 hours, and 4% reported adherence with adult guidelines. The results of this study suggest significant heterogeneity in the pediatric practice and calls for large scale studies to further develop guidelines for the pediatric population.

There is a sparsity of data and no targeted guidelines for management of acute cholecystitis in the pediatric population. The purpose of this study was to determine outcomes and readmission rates among pediatric patients with cholecystitis who underwent index cholecystectomy versus those who did not. We hypothesize that delaying cholecystectomy in pediatric patients would increase length of stay and readmission rates, similar to the studies in the adult population.

## Materials and methods

### Data source and study design

This is a retrospective cohort study of admissions to US acute-care hospitals acute cholecystitis among children and young adults. Data on hospital admissions of all pediatric patients (age < 18 years) in 2018 was extracted from the National Readmissions Database (NRD). NRD is an inpatient database with several key features: it ‘provides sufficient data for analysis across hospital types and the study of readmissions for relatively uncommon disorders and procedures, [contains] discharge data from 27 geographically dispersed states, accounting for 57.8% of the total US resident population and 56.6% of all US hospitalizations, is designed to support national readmission analyses, and cannot be used for regional, state-, or hospital-specific analyses.’ Of note, this database links patient readmission to same or any other hospital in the USA for each calendar year (1 January through 31 December) but does not link patient data across preceding or subsequent years. Therefore, we excluded index admissions occurring in December from our analysis since readmissions for those encounters could not be tracked. This study was exempted from IRB review, as no identifiable patient data were included in the database.

### Study population

We used *International Classification of Diseases, Tenth Revision, Clinical Modification* (ICD-10-CM) codes to identify all hospitalized pediatric patients (age < 18 years) with a primary diagnosis of acute cholecystitis (K80.00, K80.01, K80.12, K80.13, K81.0) and who survived to hospital discharge. All diagnostic and procedural codes used for classifications are shown in Supplementary Table 1. Admissions were identified by querying for all diagnostic ICD-10-CM codes corresponding to acute cholecystitis.

### Definitions of variables

The NRD collects demographic information, including age, sex, income, and primary and secondary insurance for all admitted patients. It also contains hospital information (e.g. bed size, location, and teaching status). The All Patients Refined Diagnosis Related Groups (APR-DRG) severity score is also recorded in the NRD and was used to assess symptom severity. The APR-DRG scores are calculated from discharge billing codes and are based on primary and secondary discharge diagnosis, age, and preexisting medical conditions. Use of these scores have been been shown to be an accurate assessment of symptom severity in the pediatric population [12, 13]. The NRD definition of hospital size is available in Supplementary Table 2.

### Outcomes

The primary outcome of this study was the rate of readmission in patients admitted with acute cholecystitis to US hospitals based on index or delayed admission cholecystectomy. We performed a multivariate regression analysis to identify predictors of all-cause 30-day readmission in these patients.

### Statistical analysis

Data are presented as raw number (*n*) and weight frequency (%) for categorical variables or mean and standard deviation (SD) for continuous variables. A univariate analysis was first performed to assess differences between the two groups (no cholecystectomy during index admission vs performance of the procedure during index admission); continuous variables were compared by using *t*-tests and categorical variables were compared by using χ^2^ tests. Univariate logistic analysis was used to identify factors with *p* < 0.10 to be considered in multivariable regression. Backward selection was employed using *p* < 0.05 for retention into the final model. Multivariable logistic analysis was used to assess differences between the groups in terms of the outcomes of interest while adjusting for patient and hospital characteristics. The NRD is based on a complex sampling design that includes stratification, clustering, and weighting. Results from the multivariable analyses were represented using odds ratios (OR) and 95% confidence intervals. *p*-values < 0.05 were considered statistically significant. Model performance was confirmed with both Pearson and Hosmer–Lemeshow goodness-of-fit testing. All analyses were performed with STATA (Version 17, College Station, TX).

## Results

We identified 550 unique admissions in pediatric patients with a diagnosis of acute cholecystitis. Demographic and baseline characteristics are presented in Table 1. A majority of patients were female (*n* = 371, 67.6%) A majority of admissions were in large (65.1%), not-for-profit, private (74%) metropolitan teaching hospitals (81.3%). Most patients had a length of stay ≤ 7 days (*n* = 496, 90.2%), Medicaid insurance (*n* = 354, 64.6%), and median household incomes in the 0–25% percentile (*n* = 186, 34.1%). The readmission rate for the entire cohort was 6.9%.

**Table 1:** Baseline characteristics of patients with cholecystitis, by cholecystectomy status

	Overall (*n* = 550)	Stratified by cholecystectomy status		*p*-value
	*n*, %	No cholecystectomy (*n* = 115)	Cholecystectomy (*n* = 435)	
*Sex*				0.53
Female	372 (67.6%)	75 (65.2%)	297 (68.3%)	
*Age*	14.6 (3.0)	13.3 (4.2)	14.9 (2.5)	< 0.001
*Weekend admission*	144 (26.2%)	29 (25.2%)	115 (26.4%)	0.79
*Hospital size*				0.82
Small	90 (16.4%)	17 (14.8%)	73 (16.8%)	
Medium	102 (18.6%)	23 (20.0%)	79 (18.2%)	
Large	358 (65.1%)	75 (65.2%)	283 (65.1%)	
*Hospital type*				0.32
Government	100 (18.2%)	25 (21.7%)	75 (17.2%)	
Private, not-for-profit	407 (74.0%)	84 (73.0%)	323 (74.3%)	
Private, profit	43 (7.8%)	6 (5.2%)	37 (8.5%)	
*Hospital location and teaching status*				0.033
Metropolitan non-teaching	67 (12.2%)	6 (5.2%)	61 (14.0%)	
Metropolitan teaching	447 (81.3%)	102 (88.7%)	345 (79.3%)	
Nonmetropolitan hospital	36 (6.5%)	7 (6.1%)	29 (6.7%)	
*Length of stay*				< 0.001
LOS ≤ 7 days	496 (90.2%)	89 (77.4%)	407 (93.6%)	
LOS > 7 days	54 (9.8%)	26 (22.6%)	28 (6.4%)	
*Payer*				0.17
Medicare	5 (0.91%)	2 (1.7%)	3 (0.7%)	
Medicaid	354 (64.6%)	79 (68.7%)	275 (63.5%)	
Private insurance	153 (27.9%)	31 (27.0%)	122 (28.2%)	
Self-pay/other	36 (6.6%)	3 (2.6%)	33 (7.6%)	
*Median household income*				0.063
0-25th percentile	186 (34.1%)	45 (39.5%)	141 (32.6%)	
26-50th percentile	158 (28.9%)	35 (30.7%)	123 (28.5%)	
51-75th percentile	111 (20.3%)	13 (11.4%)	98 (22.7%)	
76-100th percentile	91 (16.7%)	21 (18.4%)	70 (16.2%)	
*APR-DRG severity*				< 0.001
Minor	258 (46.9%)	23 (20.0%)	235 (54.0%)	
Moderate	169 (30.7%)	45 (39.1%)	124 (28.5%)	
Major	94 (17.1)	26 (22.6%)	68 (15.6%)	
Extreme	29 (5.3%)	21 (18.3%)	8 (1.84%)	
*Readmission rate*	38 (6.9%)	26 (22.6%)	12 (2.8%)	< 0.001

Data are presented as mean (SD) for continuous measures, and *n* (%) for categorical measures

*Χ*^2^ testing conducted for categorical variables, while Student *T*-Test conducted for continuous variables

## Cholecystectomy vs no cholecystectomy

Cholecystectomy was performed in 435 (79.1%) patients on index hospitalization (Table 1). Patients who received a cholecystectomy had higher mean age than those who did not (14.9 vs 13.3, *p* < 0.001). Patients who did not undergo an index cholecystectomy were more likely to have a length of stay > 7 days (22.6% vs 6.4%, *p* < 0.001) and had a significantly higher 30-day readmission rate (22.6% vs 2.8%, *p* < 0.001). There was no significant difference in rates of weekend admissions, hospital size, hospital type, hospital location, or median household income between the two groups.

## Predictors of 30-day readmission

Table 2 shows univariable and multivariable analysis of predictors of 30-day readmissions. On univariable analysis female sex (OR 2.7, CI 1.11–6.58, *p* = 0.029), length of stay ≥ 7 days (OR 3.80, CI 1.73–8.33, *p* = 0.001), APR-DRG severity category of severe disease (OR 7.14, CI 2.51–20.28, *p* < 0.001), and not having an index cholecystectomy (OR 10.30, CI 5.00–21.20, *p* < 0.001) were associated significantly increased odds of 30-day readmission. On multivariate analysis, female sex (OR 3.37, CI 1.31–8.69, *p* = 0.012) or patients who did not undergo index cholecystectomy (OR 10.66, 95% CI 5.06–22.45, *p* < 0.001) had a significantly higher odds of 30-day readmission.

**Table 2:** Predictors of 30-day readmission for patients with acute cholecystitis

Predictors	Univariate regression		Multivariate logistic regression	
	Odds ratio (95% CI)	*p*-value	Odds Ratio (95% CI)	*p*-value
*Sex*				
Male	Reference	Reference	Reference	Reference
Female	2.70 (1.11–6.58)	0.029	3.37 (1.31–8.69)	0.012
*Age*	0.93 (0.86–1.00)	0.054	0.97 (0.88–1.08)	0.609
*Weekend admission*				
No	Reference	Reference	^*^	^*^
Yes	0.87 (0.40–1.88)	0.717	^*^	^*^
Hospital size*				
Small	Reference	Reference	^*^	^*^
Medium	0.48 (0.14–1.71)	0.260	^*^	^*^
Large	0.97 (0.41–2.30)	0.940	^*^	^*^
*Hospital type*				
Government	Reference	Reference	Reference	Reference
Private, not-for-profit	0.72 (0.33–1.58)	0.411	^*^	^*^
Private, profit	0.49 (0.10–2.39)	0.380	^*^	^*^
*Hospital location and teaching status*				
Nonmetropolitan hospital	Reference	Reference	Reference	Reference
Metropolitan non-teaching	0.34 (0.05–2.13)	0.248	^*^	^*^
Metropolitan teaching	0.88 (0.25–3.01)	0.835	^*^	^*^
*Length of Stay*				
< 7 days	Reference	Reference	Reference	Reference
≥ 7 days	3.80 (1.73–8.33)	0.001	**	**
*Payer*				
Medicaid	Reference	Reference	Reference	Reference
Medicare	N/A	N/A	^**^	^**^
Private insurance	1.02 (0.49–2.13)	0.05	^**^	^**^
Self-pay/other	0.77 (0.18–3.42)	0.735	^**^	^**^
*Median household income*				
0-25th percentile	Reference	Reference	Reference	Reference
26-50th percentile	1.07 (0.44–2.60)	0.873	^*^	^*^
51-75th percentile	1.40 (0.56–3.50)	0.468	^*^	^*^
76-100th percentile	1.53 (0.59–3.96)	0.377	^*^	^*^
*APR-DRG Severity*				
Minor	Reference	Reference	Reference	Reference
Moderate	1.87 (0.82–4.28)	0.138	^**^	^**^
Major	1.81 (0.68–4.81)	0.236	^**^	^**^
Severe	7.14 (2.52–20.28)	< 0.001	^**^	^**^
*Cholecystectomy performed*				
Yes	N/A	N/A	Reference	Reference
No	10.30 (5.00–21.20)	< 0.001	10.66 (5.06–22.45)	< 0.001

Multivariable logistic regression model: threshold for entry into the multivariable model was *p* < 0.1 in univariate model. Threshold for retention in the final model was *p* < 0.05. Hospital size was determined by NRD criteria specified by hospital location and teaching status

*Not included in multivariable model

**Not retained in the final multivariable model

## Discussion

In this retrospective study using a large national database, we found that 20.9% of pediatric patients with acute cholecystitis did not receive a cholecystectomy during their index admission. These patients had a significantly higher readmission rate, which was more than ten times higher than those patients who received an index cholecystectomy. This study is one of the largest pediatric cohorts comparing outcomes and readmission rates in patients who received or did not receive cholecystectomy on index admission for acute cholecystitis and adds to the paucity of data available. Indeed, there are only few studies in the literature and no current pediatric guidelines [10–14]. The significant benefit of index cholecystectomy in pediatric patients should be highlighted in future guidelines on this topic.

The Cochrane Database of systematic reviews published a review in 2013 with a meta-analysis evaluating early versus delayed laparoscopic cholecystectomy in adult patients with acute cholecystitis from gallstone disease. The review included a total of 488 patients in six trials, and found decreased hospital stay and risk of emergency surgery without a significant difference in surgical complication rates such as bile duct injury [15]. A study from 2019 including 109,862 cholecystectomies for acute cholecystitis in adults similarly showed early cholecystectomy (< 72 h) was associated with fewer complications, lower length of stay, and fewer 30-day readmission and ED visits [15]. Although optimal timing of cholecystectomy from symptom onset is still debated, there is consensus in the adult guidelines that cholecystectomy is recommended on initial hospitalization in patients with acute cholecystitis. [5–7, 16] Conversely, there are no clear guidelines on the timing of cholecystectomy in pediatric patients with acute cholecystitis, so practices are varied with an observed preferred conservative approach in the pediatric population [10]. Our study shows similar results with the adult population in decreasing length of stay and fewer 30-day readmission rates with pediatric patients who underwent index cholecystectomy for acute cholecystitis.

Several studies have shown that the conservative approach for pediatric patients with gallstone disease is associated with increased readmissions for complications. A multicenter randomized prospective study by Gao, et al. evaluated early cholecystectomy versus non-operative management for pediatric patients with biliary colic [17]. This study found that those who underwent conservative management were more likely to have acute readmissions for complications (19.2% vs 5%, *p* = 0.001), with recurrent cholecystitis as the most likely complication (9.2% vs 0%, *p* < 0.001). It also showed that early cholecystectomy could be performed safely within this population. Similarly, a multicenter retrospective study by Abraham et al., evaluated the impact of index admission cholecystectomy in pediatric gallstone pancreatitis [11]. This study also found higher readmission rates in the delayed group compared to the early cholecystectomy group (40% vs 5%, *p* = 0.0001), with higher recurrence rates (28% vs 2%, *p* = 0.0001). A similar study on pediatric gallstone pancreatitis showed similar readmission rates of 31% in those without early cholecystectomy [18]. Our study showed similar differences in readmission rates in the pediatric patients who underwent conservative vs early cholecystectomy specifically for acute cholecystitis (22.6% vs 2.8%, *p* < 0.001). Again, this further suggests a significant benefit in early cholecystectomy in pediatric acute cholecystitis.

In our analysis, we did not identify insurance status, age, or length of hospital stay as independent predictors of 30-day admission rate. In lieu of analyzing associated comorbidities, which in addition to being uncommon in pediatrics, would also not provide a definitive contraindication to surgery, we chose to include the APR-DRG score to serve as a proxy of disease severity to ensure no confounding by severity. Interestingly, although the “severe” disease category was associated with higher odds of readmission on univariate analysis, no level of disease severity was associated with higher rate of readmission in multivariate analysis. To provide a more granular understanding of the data, of the patients with “severe” disease, no patient who underwent index cholecystectomy was readmitted, while 50% of those who did not undergo index cholecystectomy were readmitted. Prior studies have shown that socioeconomic status in the pediatric population may not be associated with morbidity and mortality as much as racial disparities, which was not evaluated in our study [19]. Determinants of length of hospital stay in pediatric patients with gallbladder disease have been studied previously; several factors have been associated with increased length of stay including admission to children’s hospitals, elective admissions, and patients having sickle cell anemia, choledocholithiasis, or pancreatitis [20]. Our study did not further stratify patients within these groups, though we did include APR-DRG severity score in the analysis.

This is one of the largest pediatric cohorts evaluating outcomes of index cholecystectomy in patients with acute cholecystitis. While there have been several studies evaluating gallbladder disease and gallstone pancreatitis in this population, this is one of the first studies looking specifically at outcomes in acute cholecystitis. There are several limitations to this study. First, this is a retrospective study, which can demonstrate association but not causality. Due to the limitation of the database used to identify a cohort, we were unable to ensure baseline homogeneity in the population. Additionally, we did not have access to surgical techniques, outcomes, and adverse events to examine safety of index cholecystectomy. Given the nature of the study, we were unable to identify the medical decisions behind why cholecystectomy was not performed on index admission or length of stay was prolonged. We also could not evaluate social risk factors for readmission. Finally, the use of a national database that is reliant on ICD-10 coding can include coding errors or omissions in the database.

In conclusion, this study shows that early cholecystectomy leads to lower readmission rates for pediatric patients admitted for acute cholecystitis, similar to studies in adults with acute cholecystitis and other gallstone disease in the pediatric population. Additional studies will need to be performed in the pediatric population to further delineate optimal timing and safety of cholecystectomy in acute cholecystitis. However, given the marked dramatic effect on readmission, we assert that cholecystectomy should be strongly considered during the index admission for patients with acute cholecystitis.

### Supplementary Information

Below is the link to the electronic supplementary material.

Supplementary file1 (DOCX 15 KB)
