# Supplementary material for: Index admission cholecystectomy for acute cholecystitis reduces 30-day readmission rates in pediatric patients
Source: Surg Endosc. 2023 Dec 19;38(3):1351–7. doi: 10.1007/s00464-023-10632-7 (PMC10881756; doi:10.1007/s00464-023-10632-7)
Supplement: Supplementary file 1 — Supplementary file1 (DOCX 15 KB) [file 464_2023_10632_MOESM1_ESM.docx]

| Supplemental Table 1: Pancreatic and Biliary Disorder Diagnosis Codes (ICD-10) | | | |
| --- | --- | --- | --- |
| Cholecystitis ICD Codes | ICD-10 Codes | Surgery Procedure Codes | ICD-10 Codes |
| Acute Cholecystitis | K80.00, K80.01, K80.12, K80.13, K81.00 | **Laproscopic cholecystectomy (open and laproscopic technique)** | 0FT40ZZ, 0FT44ZZ |

| **Supplemental Table 2: BEDSIZE CATEGORIES (Beginning in 1998)** | | | |
| --- | --- | --- | --- |
| Location and Teaching Status | **Hospital Bedsize** | | |
|  | Small | Medium | Large |
| **NORTHEAST REGION** | | | |
| Rural | 1-49 | 50-99 | 100+ |
| Urban, nonteaching | 1-124 | 125-199 | 200+ |
| Urban, teaching | 1-249 | 250-424 | 425+ |
| **MIDWEST REGION** | | | |
| Rural | 1-29 | 30-49 | 50+ |
| Urban, nonteaching | 1-74 | 75-174 | 175+ |
| Urban, teaching | 1-249 | 250-374 | 375+ |
| **SOUTHERN REGION** | | | |
| Rural | 1-39 | 40-74 | 75+ |
| Urban, nonteaching | 1-99 | 100-199 | 200+ |
| Urban, teaching | 1-249 | 250-449 | 450+ |
| **WESTERN REGION** | | | |
| Rural | 1-24 | 25-44 | 45+ |
| Urban, nonteaching | 1-99 | 100-174 | 175+ |
| Urban, teaching | 1-199 | 200-324 | 325+ |

HCUP NRD Description of Data Elements. Healthcare Cost and Utilization Project (HCUP). August 2015. Agency for Healthcare Research and Quality, Rockville, MD. www.hcup-us.ahrq.gov/db/vars/hosp_bedsize/nrdnote.jsp.
